# *APOE *ε4 lowers age at onset and is a high risk factor for Alzheimer's disease; A case control study from central Norway

**DOI:** 10.1186/1471-2377-8-9

**Published:** 2008-04-16

**Authors:** Sigrid B Sando, Stacey Melquist, Ashley Cannon, Michael L Hutton, Olav Sletvold, Ingvild Saltvedt, Linda R White, Stian Lydersen, Jan O Aasly

**Affiliations:** 1 Department of Neuroscience, Norwegian University of Science and Technology, (NTNU), Trondheim, Norway; 2 Department of Neurology, St Olav's Hospital, Trondheim, Norway; 3 Department of Neuroscience, Mayo Clinic College of Medicine, Jacksonville, Florida, USA; 4 Unit for Applied Clinical Research, Department of Cancer Research and Molecular Medicine, The Norwegian University of Science and Technology (NTNU), Trondheim, Norway; 5 Department of Geriatric medicine, St Olav's Hospital, Trondheim, Norway

## Abstract

**Background:**

The objective of this study was to analyze factors influencing the risk and timing of Alzheimer's disease (AD) in central Norway. The *APOE *ε4 allele is the only consistently identified risk factor for late onset Alzheimer's disease (LOAD). We have described the allele frequencies of the apolipoprotein E gene (*APOE*) in a large population of patients with AD compared to the frequencies in a cognitively-normal control group, and estimated the effect of the *APOE *ε4 allele on the risk and the age at onset of AD in this population.

**Methods:**

376 patients diagnosed with AD and 561 cognitively-normal control individuals with no known first degree relatives with dementia were genotyped for the *APOE *alleles. Allele frequencies and genotypes in patients and control individuals were compared. Odds Ratio for developing AD in different genotypes was calculated.

**Results:**

Odds Ratio (OR) for developing AD was significantly increased in carriers of the *APOE *ε4 allele compared to individuals with the *APOE *ε3/ε3 genotype. Individuals carrying *APOE *ε4/ε4 had OR of 12.9 for developing AD, while carriers of *APOE *ε2/ε4 and *APOE *ε3/ε4 had OR of 3.2 and 4.2 respectively. The effect of the *APOE *ε4 allele was weaker with increasing age. Carrying the *APOE *ε2 allele showed no significant protective effect against AD and did not influence age at onset of the disease. Onset in LOAD patients was significantly reduced in a dose dependent manner from 78.4 years in patients without the *APOE *ε4 allele, to 75.3 in carriers of one *APOE *ε4 allele and 72.9 in carriers of two *APOE *ε4 alleles. Age at onset in early onset AD (EOAD) was not influenced by *APOE *ε4 alleles.

**Conclusion:**

*APOE *ε4 is a very strong risk factor for AD in the population of central Norway, and lowers age at onset of LOAD significantly.

## Background

Apolipoprotein E (APOE) is a multifunctional circulating lipoprotein consisting of 299 amino acids, synthesized in various organs, including liver, spleen, kidney and brain [1]. Three common isoforms of the protein are identified as ApoE 2, ApoE 3 and ApoE 4. These isoforms are coded for by three different alleles (denoted by a specific combination of two coding SNPs), located within the *APOE *gene on chromosome 19q13.2. *APOE *ε2, *APOE *ε3 and *APOE *ε4 account for more than 99% of the variation of *APOE *[2]. The frequencies of these three *APOE *alleles are highly variable in different populations [3-5]. Globally, *APOE *ε3 is the most frequently occurring allele, constituting 60–90% of the allelic variation. *APOE *ε2 constitutes 0–20% and *APOE *ε4 10–20% of allelic variation [5,6]. *APOE *ε3 and *APOE *ε4 are also found to be inversely correlated in European populations with the *APOE *ε4 allele found more frequently in populations in northern Europe than in southern Europe [4].

Carrying the *APOE *ε4 allele is a risk factor for early onset Alzheimer's disease (AD), and is the only consistently identified risk factor for late-onset Alzheimer's disease (LOAD) [7-11]. *APOE *ε4 acts in a dose-dependant manner: carriers of two *APOE *ε4 alleles have a higher risk and earlier onset of AD than heterozygous subjects [12-14].

Only a few studies have examined the *APOE *allele frequencies in AD patients in Scandinavia [15-19]. In the present study we describe *APOE *genotypes and allele frequencies in AD patients and a cohort of controls from central Norway where the population is comparatively genetically homogeneous.

## Methods

The clinical material consisted of 376 patients diagnosed with probable or possible AD and 561 cognitively-normal controls, all ethnic Norwegians. The patients were neurological and geriatric patients recruited from the University Hospital of Trondheim, the district hospital in Namsos and patients from nursing homes in central Norway. The inclusion period lasted from May 2003 until September 2006. Patients or suitable proxies were asked about case history, including a family history of dementia. Neurological examination was completed by one neurologist (SBS) in all patients and included the Mini-Mental State Examination (MMSE) [20] and Clock Drawing Test [21]. Blood screening was performed, and secondary causes of dementia were excluded. Additional tests including EEG, lumbar puncture or an olfactory test were performed in subgroups [22]. Blood samples for DNA extraction and genetic testing were obtained from all patients and control subjects.

The guidelines given in the International Classification of Diseases tenth revision (ICD-10) were applied for diagnosing dementia. Patients diagnosed with Alzheimer's disease met the Criteria for probable or possible AD from the National Institute of Neurological and Communicative Disorders and Stroke and the Alzheimer's disease and Related Disorders Association (NINCDS-ADRDA) [23].

Healthy spouses and members of societies for retired people in central Norway were recruited for the cognitively-normal control group. Only ethnic Norwegians without known dementia in first degree relatives were included. Both patients and controls were asked for place of birth, place of residence during childhood, number of years of education, and former occupation. Informed consent was obtained from patients or suitable proxies and from all control subjects. The study was approved by the Regional Committee for Medical Research Ethics in central Norway.

### Genotyping

Overall APOE genotype was determined by the combinations of genotypes at two single nucleotide polymorphisms (SNPs), rs7412 and rs429358. Genotyping was performed using predesigned Taqman Assays on the Applied Biosystems 7900 HT Fast Real Time PCR system, and genotype calls were made using the SDS v2.2 software (Applied Biosystems). Any sample in which the Taqman assays gave inconclusive allele calls was re-genotyped using a restriction fragment length polymorphism (RFLP) method [24]. Deviation from Hardy Weinberg Equilibrium (HWE) was calculated for the APOE locus, and both cases (p = 0.86) and controls (p = 0.36) were found to be in HWE.

### Statistical analyses

Data analysis was chiefly performed with the SPSS, version 13. Categorical variables were compared using Pearson's chi-square test. Age at onset in different groups was compared by Wilcoxon-Mann-Whitney's test. Odds ratios (OR) were calculated for each genotype by binary logistic regression, using the ε3/ε3 genotype as reference value. Logistic regression was performed with exact conditional maximum likelihood and median unbiased estimation in LogXact. Two-sided p-value < 0.05 was considered significant. Standard deviation (SD) is given after mean value as ± SD.

## Results

264 patients were diagnosed with probable AD (70.2%), and 112 with possible AD (29.8%) according to the NINCDS-ADRDA criteria. 263 were women (69.9%) and 113 were men (30.1%). 213 of the patients had first degree relatives with dementia (56.6%), and 149 (39.6%) had no known dementia in parents, siblings or children. Family history was missing in 14 patients. Mean age at inclusion for patients was 79.5 ± 8.2 years. The median MMSE score was 17 (interquartile range 11–22). MMSE scores were missing in four patients.

The control group consisted of 561 subjects; 338 women (60.2%) and 223 men (39.8%) (Table 1). Age at inclusion for control individuals was 75.1 ± 7.3 years.

**Table 1:** Number of patients, age at inclusion, allele frequencies and genotypes in patients and controls

	Number (Females/Males)	Age at inclusion ± SD	Allele frequencies (%)			Genotypes					
			
			ε2	ε3	ε4	ε2/ε2	ε2/ε3	ε2/ε4	ε3/ε3	ε3/ε4	ε4/ε4
Alzheimer patients	All 376	79.5 ± 8.2	7.3	53.1	39.6	0.3	7.4	6.6	28.2	42.3	15.2
	F 263	80.3 ± 8.3	7.6	52.9	39.5	0.4	6.8	7.6	28.5	41.8	14.8
	M 113	77.5 ± 7.6	6.6	53.5	39.8	0.0	8.8	4.4	27.4	43.4	15.9

Control persons	All 561	75.1 ± 7.3	11.3	74.3	14.3	0.7	17.1	4.1	55.8	20.0	2.3
	F 338	74.7 ± 7.1	10.9	74.4	14.3	0.6	16.6	4.1	55.9	21.0	1.8
	M 223	75.7 ± 7.5	11.9	73.8	14.3	0.9	17.9	4.0	55.6	18.4	3.1

F = females

M = males

SD = Standard Deviation

Allele frequencies and genotypes were calculated for patients and controls, and for gender in each group (Table 1). The allele frequencies for men and women did not differ significantly either in controls or patients. The differences in allele frequencies in patients and controls were significant. Allele frequencies were also calculated for patients and control individuals according to age (Table 2). The frequency of the *APOE *ε4 allele in patients was highest in those with age at onset 60–69 years (51.4%). The oldest patients with onset ≥ 80 years had the lowest proportion of the *APOE *ε4 allele (24.8%). While 64.1% (n = 241) of the AD patients had one or two *APOE *ε4 alleles, only 26.4% (n = 148) of the control individuals carried an *APOE *ε4 allele (p < 0.001). In patients with dementia in first degree relatives 70.9% (n = 151) had one or two *APOE *ε4 alleles, compared to 56.4% (n = 84) in patients with no known history of dementia in first degree relatives (p = 0.004).

**Table 2:** Allele frequencies by age

	Age	Number	Allele frequencies(%)		
			ε2	ε3	ε4
	< 60	24	8.3	52.1	39.6
Alzheimer patients	60–69	74	6.8	41.9	51.4
Age at onset	70–79	169	5.6	50.3	44.1
	80–89	109	10.1	65.1	24.8
	< 60	13	11.5	65.4	23.1
Control persons	60–69	109	11.0	72.9	16.1
Age at inclusion	70–79	293	10.6	76.1	13.3
	80–89	135	12.6	72.2	15.2
	90–99	11	18.2	77.3	4.5

The odds ratios (OR) for developing AD were calculated for each *APOE *genotype, using ε3/ε3 as the reference value (Table 3). These analyses were also carried out separately for patients with and without dementia in first degree relatives. Significantly increased ORs were found in all genotypes containing the *APOE *ε4 allele, both for the group with dementia in first degree relatives and for those with a negative family history. Odds Ratios for genotypes containing an *APOE *ε4 allele were increased in the group with a positive family history compared to those without dementia in first degree relatives. Logistic regression with the number of *APOE *ε2 and *APOE *ε4 alleles as covariates showed no significant protective effect of either one *APOE *ε2 allele (OR = 0.82; p = 0.32) or two *APOE *ε2 alleles (OR = 0.73; p = 0.78). Logistic regression analysis with *APOE *ε4 and age as covariates, showed a non-significant (p = 0.14) interaction, with a reduced effect of *APOE *ε4 in the older patients.

**Table 3:** Odds Ratio for AD in all genotypes in patients with/without first degree relatives with dementia

	Genotypes					
	
	ε2/ε2	ε2/ε3	ε2/ε4	ε3/ε3	ε3/ε4	ε4/ε4
Odds Ratio (376 AD patients; 561 control individuals)	0.7	0.9	**3.2**	1	**4.2**	**12.9**
CI	0.01–7.57	0.51–1.41	1.67–6.18	Ref	2.98–5.89	6.64–26.68
p value	1.00	0.62	< 0.001		< 0.001	< 0.001

Odds Ratio, AD patients with no family history of dementia (n = 149)	1.1*	0.7	**2.8**	1	**2.9**	**8.1**
CI	0.00–0.16	0.34–1.47	1.17–6.44	Ref	1.83–4.58	3.53–19.18
p value	1.00	0.47	0.021		< 0.001	< 0.000

Odds Ratio, AD patients with first degree relative with dementia (n = 213)	1.7	1.0	**4.0**	1	**5.9**	**18.7**
CI	0.03–17.27	0.47–1.89	1.79–8.86	Ref	3.88–9.15	8.97–41.40
p value	1.0	1.0	< 0.001		< 0.001	< 0.001

SD = Standard Deviation

CI = 95% confidence interval

Ref = Reference category

*The Median unbiased estimate is given, since the Maximum Likelihood estimate does not exist.

**Figure 1:**
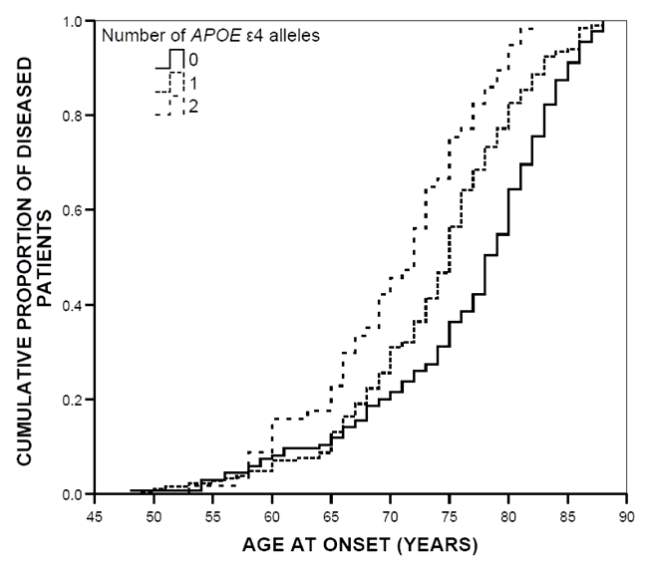
Cumulative proportion of diseased patients.

Age at onset in patients with LOAD was significantly reduced by the *APOE *ε4 allele in a dose dependent manner, while it had no lowering effect in patients with onset before 65 years (early onset AD, EOAD) (Table 4). In LOAD patients without the *APOE *ε4 allele, mean age at onset was 78.4 years, whereas those carrying one *APOE *ε4 allele had onset at 75.3 years (p = 0.005). For patients with two *APOE *ε4 alleles, age at onset was further reduced to 72.9 years. The difference in onset between carriers of one and two *APOE *ε4 alleles was also significant (p = 0.002). Figure 1 shows onset in carriers of zero, one and two *APOE *ε4 alleles. The presence of an *APOE *ε2 allele had no significant effect on age at onset.

**Table 4:** Effect of the *APOE *ε4 allele on age at onset in LOAD and EOAD

	Number of *APOE *ε4-alleles					
	
	**0**		**1**		**2**	
EOAD. Age at onset ± SD, n	57.3 ± 4.0	14	57.3 ± 4.7	16	58.7 ± 2.6	10
LOAD. Age at onset ± SD, n	78.4 ± 5.8	121	75.3 ± 5.8^a^	168	72.9 ± 5.0^b^	47

SD = Standard deviation

n = number of patients

EOAD: Early onset Alzheimer's disease (onset < 65 years), no significant difference in onset between carriers of none, one and two *APOE *ε4 alleles.

LOAD: Late onset Alzheimer's disease (onset ≥ 65 years)

^a^: The difference between onset in LOAD (Late Onset AD) in carriers of none and one *APOE *ε4 allele was significant; p = 0.005.

^b^: The difference between onset in LOAD in carriers of one and two *APOE *ε4 alleles was significant; p = 0.002.

## Discussion

In this study we have described the *APOE *allele frequencies in 376 AD patients and 561 healthy controls from central Norway. Individuals carrying the *APOE *ε4 allele had an increased OR for developing AD, both in homozygous (12.9) and heterozygous individuals (4.2 for ε3/ε4, 3.2 for ε2/ε4). Age at onset in LOAD patients was significantly lowered by about three years for every *APOE *ε4 allele the patient carried. The *APOE *ε2 allele did not affect age at onset of AD and had no significant protective effect on the risk of AD.

While smaller studies in this field have been published from Norway, to our knowledge this is the first report of *APOE *allele frequencies and *APOE *genotypes in a large Norwegian AD population and the largest study on *APOE *allele frequencies in AD patients from Scandinavia to date. The study was performed in central Norway where the migration of the population for several centuries has been very modest. The population is therefore quite genetically homogeneous. The frequency of *APOE *ε4 in patients in the present study was similar to the result obtained in a large meta-analysis of 5107 Caucasian AD patients, where the *APOE *ε4 frequency was found to be 36.7% [25]. Age dependent variation in frequency of the *APOE *ε4 allele in AD patients has been reported earlier [26] and was also present in our study. In North America, the estimated *APOE *ε4 allele frequency is 60% in the AD population (15% ε4/4 and 40% with ε3/4 and < 5% with ε2/4) [27]. In our study, we found that 64.1% of the patients carried one or more *APOE *ε4 alleles (15.2% had ε4/4, 42.3% had ε3/ε4 and 6.6% had ε2/4). Our findings are thus in keeping with previous estimates in Caucasians.

The frequency of *APOE *ε4 in control individuals in the present study was also similar to the frequency in a meta-analysis of 6262 Caucasian control subjects from 1997 (13.7%) [25], though less than previously reported in a Norwegian study (19.8%) [28]. However, the latter study was conducted in 798 healthy blood donors aged < 40 years. A decrease in the frequency of the *APOE *ε4 allele with increasing age has been reported in healthy individuals [29]. As our control population was significantly younger than the AD patients, the *APOE *ε4 frequency might actually be slightly increased compared to controls of the same age as the patients. However, only control individuals without known dementia in first degree relatives were included in the present study, to increase the probability of detecting genetic differences between AD patients and control individuals. This selection may have caused a lower proportion of the *APOE *ε4 allele than in an unselected control population, as earlier findings suggest that offspring of AD patients have a frequency of the *APOE *ε4 allele that exceeds population estimates [30]. The frequency of patients reporting dementia in one or more first degree relatives was high in this population (56.6%). In most cases, both patients and next of kin were asked about relatives with cognitive impairment, and this may have increased the trend to report family members with dementia. Other studies have also reported a high occurrence of AD patients with dementia in first degree relatives [19,31].

The present study confirmed that individuals carrying the *APOE *ε4 allele are at increased risk for developing AD. The ORs found in our study was close to those found previously in a large meta-analysis [25]. As the ORs calculated in the present study may slightly overestimate the effect of the *APOE *ε4 allele because of selection of the control group, we performed analyses of the population of patients with and without first degree relatives separately, using the same 561 selected controls for both analyses. This calculation showed increased ORs in the group consisting of patients with dementia in first degree relatives compared to the group without a known family history of dementia. The true estimate of OR is in between these estimates.

Age at onset in LOAD was strongly influenced by the number of *APOE *ε4 alleles in this study, and decreased by about three years for every *APOE *ε4 allele the patient carried. The decrease in age at onset of AD in carriers of the *APOE *ε4 allele is well known [12,13], though the magnitude of the decrease in onset varies. A Finnish study found that age at onset decreased from 76 to 69 years in LOAD as the number of *APOE *ε4 alleles increased from 0 to 2 [32], whereas a twin study from Norway demonstrated no effect on age at onset of the *APOE *ε4 allele, probably due to the small number of patients included [17].

Occurrence of the *APOE *ε4 allele did not influence age at onset in patients with EOAD in the present study. Similar results are reported from another study with a larger sample size [29]. The occurrence of the *APOE *ε2 allele in control individuals in the present study was 11.3%, while the frequency in AD patients was 7.3%. Both of these *APOE *ε2 frequencies are increased compared to frequencies described in a meta-analysis [25], where the frequency for control individuals was 8.4% and for patients 3.9%. The increased *APOE *ε2 frequency may be a characteristic unique to this Norwegian population, as previous populations may have been too small to observe this increase [15,17].

How the *APOE *ε4 allele exerts its influence is not fully understood, and the magnitude of the influence is also disputed. In the Framingham study [33] an increased risk for AD was found both in homozygous and heterozygous carriers of the *APOE *ε4 allele. However, most *APOE *ε4-carriers in the Framingham study did not develop AD. The authors emphasized that around half of all AD cases is not caused by *APOE *ε4. Others consider the *APOE *ε4 allele to be responsible for as much as 95% of the AD cases in North America [34]. However, not all patients with AD carry an *APOE *ε4 allele, and not all carriers of the APOE ε4 allele develop AD [35]. In the present study 35.9% of AD patients had no *APOE *ε4 allele, and 26.4% of the control population carried one or two *APOE *ε4 alleles. This confirms that the *APOE *ε4 allele is neither necessary nor sufficient for developing AD.

The incidence of AD increases by increasing age [36]. In our study, analysis of the interaction of *APOE *ε4 by age indicated that the effect of the *APOE *ε4 allele was weaker with increasing age. The frequency of the *APOE *ε4 allele decreased after 80 years in AD patients while the frequency of the *APOE *ε2 allele increased. This suggests that the reported increase in AD in individuals aged ≥ 80 years is likely due to genetic or environmental factors other than the *APOE *ε4 [36,37]. Consequently, factors influencing the risk of AD in this age group may be interesting for further studies.

The *APOE *ε4 allele is the only known genetic risk factor for LOAD. The present study demonstrates that also in this Norwegian population the *APOE *ε4 allele is a strong risk factor for dementia, similar to what is seen in other Caucasian populations. The effect of two *APOE *ε4 alleles is stronger than of one, regarding both risk and age at onset. Because of the similarities seen with respect to genetic risk of *APOE *ε4 in this Norwegian and other Caucasian populations and with the likely increased genetic homogeneity due to population demographics, this Norwegian population may serve as an ideal population to search for additional genetic risk factors contributing to risk of developing of AD.

## Competing interests

The author(s) declare that they have no competing interests.

## Authors' contributions

SBS has done the clinical and neurological evaluation of all included patients, recruited the control individuals, done some of the statistics and drafted the manuscript. AC has carried out genotyping of the samples. SM has been involved in conception and design of the study, carried out genotyping of the samples, and has revised the manuscript. IS and OS have made substantial contribution to conception and design and have recruited patients for the study. SL has made substantial work with analysing and interpretation of the data, and has been involved in revising the manuscript. JAa and LW have made important contribution to conception and design of the study and to drafting and revising the manuscript. MH has made substantial contribution to conception and design of the study. All authors have read and approved the final manuscript.

## Pre-publication history

The pre-publication history for this paper can be accessed here:


